# Electric Stimulation of Neurogenesis Improves Behavioral Recovery After Focal Ischemia in Aged Rats

**DOI:** 10.3389/fnins.2020.00732

**Published:** 2020-07-09

**Authors:** Adrian Tudor Balseanu, Monica Grigore, Leonard-Radu Pinosanu, Mark Slevin, Dirk M. Hermann, Daniela Glavan, Aurel Popa-Wagner

**Affiliations:** ^1^Center of Clinical and Experimental Medicine, Department of Psychiatry, University of Medicine and Pharmacy of Craiova, Craiova, Romania; ^2^Center of Clinical and Experimental Medicine, Doctoral School, University of Medicine and Pharmacy of Craiova, Craiova, Romania; ^3^Department of Life Sciences, Faculty of Science and Engineering, Manchester Metropolitan University, Manchester, United Kingdom; ^4^Department of Neurology the Chair of Vascular Neurology and Dementia, Essen University Hospital, Essen, Germany; ^5^Menzies Health Institute Queensland, Griffith University, Southport, QLD, Australia

**Keywords:** stroke, aging, rats, electrical stimulation, neurogenesis, behavior

## Abstract

The major aim of stroke therapies is to stimulate brain repair and to improve behavioral recuperation after cerebral ischemia. Despite remarkable advances in cell therapy for stroke, stem cell-based tissue replacement has not been achieved yet stimulating the search for alternative strategies for brain self-repair using the neurogenic zones of the brain, the dentate gyrus and the subventricular zone (SVZ). However, during aging, the potential of the hippocampus and the SVZ to generate new neuronal precursors, declines. We hypothesized that electrically stimulation of endogenous neurogenesis in aged rats could increase the odds of brain self-repair and improve behavioral recuperation after focal ischemia. Following stroke in aged animals, the rats were subjected to two sessions of electrical non-convulsive stimulation using ear-clip electrodes, at 7- and 24 days after MCAO. Animal were sacrificed after 48 days. We report that electrical stimulation (ES) stimulation of post-stroke aged rats led to an improved functional recovery of spatial long-term memory (T-maze) but not on the rotating pole or the inclined plane, both tests requiring complex sensorimotor skills. Surprisingly, ES had a detrimental effect on the asymmetric sensorimotor deficit. Histologically, there was a robust increase in the number of doublecortin-positive cells in the dentate gyrus and SVZ of the infarcted hemisphere and the presence of a considerable number of neurons expressing tubulin beta III in the infarcted area. Among the gene that were unique to ES, we noted increases in the expression of *seizure related 6 homolog like* which is one of the physiological substrate of the β-secretase BACE1 involved in the pathophysiology of the Alzheimer’s disease and *Igfbp3* and BDNF receptor mRNAs which has been shown to have a neuroprotective effect after cerebral ischemia. However, ES was associated with a long-term down regulation of cortical gene expression after stroke in aged rats suggesting that gene expression in the peri-infarcted cortical area may not be related to electrical stimulation induced-neurogenesis in the subventricular zone and hippocampus.

## Introduction

The major aim of stroke therapies is to stimulate brain repair and to improve behavioral recuperation after cerebral ischemia. However, in animal models, brain tissue replacement does not occur after focal ischemia stimulating the search for alternative strategies for brain repair and behavioral recuperation after stroke. One strategy is to stimulate the endogenous brain repair and functional recovery following ischemic insult (Jin et al., 2010). Following stroke injury in striatum, in neurogenic regions (hippocampus and subventricular zone, SVZ), vigorous neurogenesis is initiated both in young and aged animals (Arvidsson et al., 2002). Furthermore, the newly born cells were shown to be able to migrate from the SVZ to the stroke-damaged striatum, develop and differentiate into the phenotype of the neurons damaged by ischemic lesion (Arvidsson et al., 2002; Yamashita et al., 2006). However, later is has been shown that migration of SVZ-neuroblasts to the nearby striatum and corpus callosum was valid only for the 2 months old mice (Adamczak et al., 2017). Furthermore, for focal cortical stroke, the migration of neuronal precursors from the SVZ to the peri-infarcted area does occur to a lesser extent (Shimada et al., 2010).

During aging, the potential of the hippocampus and the SVZ to generate new neuronal precursors declines, but the cause of this decline is not precisely known (Capilla-Gonzalez et al., 2015). Most likely, aging reduces the neurogenic potential by disrupting the intricate cellular organization of the SVZ (Capilla-Gonzalez et al., 2014). Interestingly, while neuronal cells production declines with increasing age, the generation of oligodendroglia is not altered (Capilla-Gonzalez et al., 2014).

The production of new cells in neurogenic regions can be modulated by physical activity, enriched environment, or epileptic drugs (Buga et al., 2012; Korosi et al., 2012). It is also known that abnormal brain electrical activity results in a robust increase in the number of neuronal precursors in the adult hippocampus and subventricular zone (Parent et al., 1997). However, in remote areas such as the cortex, few of the new born cells will survive and their contribution of the survived cells to neurological recovery is questionable. Therefore, we hypothesized that stimulating neurogenesis in the subventricular zone and hippocampus after the neuroinflammation has diminished (Badan et al., 2003; Joseph et al., 2012; Sandu et al., 2015), would increase the odds of behavioral recovery. Given the maturation period of neuronal precursors in the hippocampus and subventricular zone of 24 days (Buga et al., 2012), we stimulated neurogenesis by electrical stimulation (ES) at 7 and 24 days after stroke and assessed behavioral recuperation and molecular and cellular indices of post-stroke recovery of the brain in aged rats.

## Materials and Methods

In this study we used aged male Sprague-Dawley rats (*N* = 56; 18–20 months of age; 520–600 g) kept under standard condition of temperature and humidity in the Animal House Facility with free access to food and water. The group sizes for the aged rats were larger to compensate for the post-ischemic mortality rate which was of 15%, except sham-operated rats. The control and treatment groups consisted of *N* = 18 age-matched animals. The number of sham-operated rats was of *N* = 20. All experiments were performed in accordance with “ARRIVE Guidelines for the Care and Use of Laboratory Animals,” and animal procedures were approved by the Animal Experimentation Ethics Board of the State of Mecklenburg-Vorpommern as meeting the ethical requirements of the German National Act on the Use of Experimental Animals (approval no LALLF M-V/TSD/7221.3-1.1- 040/10) and by the Institutional Animal Care and Use Committee of the Medical University of Craiova (# 87-15-11-2017).

### Randomization

Random assignment was done for (i) group assignment, (ii) surgery assignment; and (iii) treatment assignment.

### Behavioral Testing

Testing procedure involved two persons, one person who did the surgery and was in charge of handling the animals according to group assignment and another one who has tested the animals and was not aware of groups’ identity. To evaluate changes in neurological function associated with ischemia, the rats were subjected to a variety of somatosensory, motor, learning, and memory tests before and after surgery. All testing was performed from 9 to 11 a.m. Results obtained before surgery were used to define 100% functionality for each animal on each test, and functional recovery was expressed either as SCORE or as percent recovery relative to the pre-surgery baseline.

#### Rotating Pole

The rotating pole task assesses coordination and sensorimotor function in the MCAO model. Each rat was tested for its ability to cross a rotating (6 rpm) horizontal rod. The score assessment was done as previously described by our group (Buchhold et al., 2007). Briefly, the time taken for the rat to traverse the rotating pole and join a group of rats visible at the finish line was measured. The score assessment was twofold: (i) time (seconds) required to tra- verse the rotating pole and (ii) the score as follows: 0 – rat falls immediately (onto a soft surface); 1 – rat does not walk forward, but stays on the rotarod; 2 – rat walks, but falls before reaching the goal; 3 – rat traverses the rod successfully, but the limbs are used asymmetrically; 4 – the left hindlimb is used less than 50% of the time taken to traverse the rod; 5 – the rat successfully traverses the rod, but with some difficulties; 6 – no mistakes, symmetric movements.

#### Asymmetric Sensorimotor Deficit: Adhesive Tape Removal Test

We assessed the asymmetry of sensorimotor deficit of the forelimbs induced by unilateral MCAO by the adhesive tape removal test. In short, sticky patches were applied on the distal hairless parts of the forelimbs and the removal time from both limbs was measured. Three trials were done separately for each limb and the means of the values were noted. If the animal did not remove the tape within 180 s, the timer was stopped. Results are given as time needed to remove the adhesive tape from one forelimb divided by the sum of time needed to remove it from both forelimbs (Popa-Wagner et al., 2010).

#### Inclined Plane

We tested the ability of each animal to maintain its position at a given angle on an inclined plane (Buchhold et al., 2007). The relative angle at which the rat could no longer maintain its position was taken as a measure of functional impairment. This test was conducted once before surgery and daily thereafter.

#### Spatial Long-Term Memory: T-Maze

T-maze was chosen to assess retention of memory on positive reinforcement as previously described (Moldovan et al., 2010). Rats were trained for 2 weeks before MCAO surgery to find food in a maze of three consecutive junctions. We scored every error with one point, therefore a successful trial was scored as 0 and the maximum number of allowed mistakes was 3. Because the apparatus was made up of 3 T-mazes, the rats could commit 1, 2, or 3 errors in each trial. On the surgery day, an average of the last three performances was calculated and was considered as baseline.

### Surgery

Prior surgery rats were fasted overnight to reduce the blood glucose levels. After craniotomy the middle cerebral artery (MCA) was exposed and slowly lifted with a tungsten hook attached to a micromanipulator (Maerzhaeuser Precision Micro-manipulator Systems, Fine Science Tools) and electrocoagulated. Both common carotid arteries were then occluded by tightening pre-positioned thread loops for 90 min. Throughout surgery, anesthesia was maintained by spontaneous inhalation of 1–1.5% isoflurane in a mixture of 75% nitrous oxide and 25% oxygen. Body temperature was controlled at 37°C by a Homeothermic Blanket System (Harvard Apparatus). The local changes in blood flow were monitored using a laser Doppler device (Perimed, Stockholm, Sweden), and blood gasses were measured at several time points during ischemia. A decrease in laser Doppler signals to <20% of control values was considered to be successful MCA occlusion. The surgical procedure for the sham group was identical, except for the MCA electrocoagulation.

After 90 min, the common carotid arteries were re-opened. The muscle and soft tissue were replaced and the skin was closed using 5-0 nylon suture. Buprenorphine was given sc twice, every 6 h after surgery at a dose of 0.3 mg/kg for peri-operative pain relief. Moisted food was provided for the first 3 days post-surgery.

Subsequent to survival time of 48 days, the rats were deeply anesthetized with 2.5% isoflurane in 75% nitrous oxide and 25% oxygen, and perfused with neutral buffered saline followed by buffered 4% freshly depolymerized paraformaldehyde. The brain was removed, post-fixed in 4% buffered paraformaldehyde for 24 h, cryoprotected in 15% glycerol prepared in 10 mmol/l phosphate buffered saline, flash-frozen in isopentane and stored at −70°C until sectioning.

### Stimulation of Endogenous Neurogenesis by Non-convulsive Electrostimulation

At 7- and 24 days after stroke, animals were stimulated by the application of ear-clip electrodes. Ear-clip electrodes were moistured with saline and attached to the pinnae. Endogenous neurogenesis in aged rats was stimulated by a subconvulsive train (30 mA, 60 pulses/sec, 0.5-ms pulse width, 1-s duration and in total for 5-s) (Torsten et al., 2000).

The ES treatment was done under mild anesthesia (ketamine 85 mg/kg, PromAce 0.85 mg/kg).

### BrdU Labeling

To label newly generated cells, rats were given injections of bromodeoxyuridine (BrdU; 50 mg/kg body weight, i.p.; Sigma) immediately after the stimulation followed by two more injections in the next 2 days.

### Immunohistochemistry

Sections (25 μm-thick) were cut on a freezing microtome and processed for immunohistochemistry as previously described (Popa-Wagner et al., 2007, 2010). For DAB staining, sections were blocked in 3% donkey serum/10 mmol/l PBS/0.3% Tween 20, overnight at 4°C. Secondary biotinylated antibodies were raised in the donkey (Jackson ImmunoResearch Laboratories, West Grove, PA, United States). Sections were stained using the ABC Elite reagents (Vectastain Elite Kit, Vector) using 0.025% 3′,3′ diaminobenzidine (DAB) and 0.005% hydrogen peroxide as the chromogen. For BrdU detection by diaminobenzidine- (DAB) staining, free-floating sections were pre-treated with 50% formamide, 0.3 M NaCl, 10 mM sodium citrate at 65°C for 2 h, incubated in 2 M HCl at 40°C for 1 h, and rinsed in 0.1 M borate buffer (pH 8.5) at room temperature for 10 min. Sections were incubated with the rat anti-BrdU (1:2000, Serotec, United Kingdom) at 4°C for 24 h. BrdU-positive nuclei were visualized using goat anti-rat-Cy5. For phenotyping, the tissue was incubated with a rabbit anti-NeuN (1:1000, Novus Biologicals, United Kingdom) and guinea pig anti-doublecortin (1:2000; Millipore, Germany) at 4°C overnight. At the next day, sections were rinsed with PBS and incubated with Alexa Fluor^®^ 568 goat anti-rabbit IgG and Alexa Fluor^®^ 488 goat anti-guinea pig IgG. Neuronal cells expressing Tubulin beta III, sections were incubated with rabbit anti-tubulin beta III (1:5000; Abcam, United Kingdom).

### Counting of Co-localized DCX/BrdU- and Tubulin Beta III/BrdU Positive Cells

Double labeled cells were analyzed in every 10th section in the region adjacent to the scar-confined area corresponding to the formerly infarct core, hippocampus and subventricular zone as previously described (Popa-Wagner et al., 2010). To this end, a sequence of confocal counting images of 161 μm × 242 μm × 25 μm, spaced 0.1 μm apart across a 25 μm-thick section and covering 30% of the infarcted area, was taken for fluorescently labeled cells (Popa-Wagner et al., 2007). The relative mean number of double labeled cells was then calculated by multiplying the number of cells per section times 3.3 (the counting boxes that were quantitated covered one third of the area of each section) times the section interval of 10. By using this approach we were able to overcome the difficulty of cell counting in both dentate gyrus and SVZ which are extremely heavily packed with cells (Acosta et al., 2017).

### Determination of Infarct Volume by MRI

Magnetic resonance imaging (MRI) was used to visualize the infarct volume for both groups at day 48 after stroke. MRI measurements were performed on a 7-T Bruker ClinScan magnet with a 20 cm inner bore, capable of 290 mT/m in 250 μs (Bruker BioSpin MRI, Ettlingen, Germany) (Jones et al., 2012). Images were received by a 2 × 2 phased array RF coil, designed specifically for rat brain studies that was placed directly on the skull. The animals were anesthetized during imaging to minimize discomfort. Respiratory rate was monitored, and isoflurane concentrations were varied between 1.5 and 2.0% to keep the respiratory rate between 35 and 45/min. After positioning the animal’s head, T2-weighted MRI was recorded were performed with a multislice spin-echo sequence with 25 slices of 0.7 mm thickness and a matrix size 640 pixels × 640 pixels, field-of-view of 32 mm × 32 mm, a repetition time (TR) of 4,330 ms, and an echo time (TE) of 45 ms.

### Lesion Measurement Using MIPAV Software

T2WI lesion volumes were determined using the image processing software Medical Image Processing, Analysis and Visualization (MIPAV, version 3.0, National Institutes of Health, Bethesda, MD, United States). After optimal adjustment of contrast, the edge of the lesion was traced manually on each of the 25 coronal slices, which completely covered the MCA territory in all animals. The areas of hyperintensity were then summed and multiplied by the slice thickness to calculate lesion volumes as previously described (Jones et al., 2012).

### Transcriptomic Analysis

#### RNA Extraction and RNA Quality Control

After the tissue was homogenized, total RNA was extracted from microdissected tissue using TRIzol reagent (Invitrogen Life Technologies, Karlsruhe, Germany). Genomic DNA was removed using the RNeasy Plus kit (Qiagen).

#### Microarray Hybridization

Prior to sample preprocessing, RNA integrity of RNA pools was assessed with the RNA 6000 nano kit using the Bioanalyzer 2100 instrument (Agilent, Böblingen, Germany). RNA integrity numbers ranged between 6.5 and 8.2. 200 ng of each sample were processed with the whole transcript (WT) expression kit (Ambion, Darmstadt, Germany), i.e., subjected to RNA amplification via reverse transcription to double-stranded cDNA and subsequent *in vitro* transcription; this was followed by another round of reverse transcription yielding single-stranded DNA in sense orientation. Hybridization cocktails were produced after fragmentation and biotin labeling of target DNAs following the protocol of the GeneChip WT terminal labeling kit (Affymetrix, Santa Clara, CA, United States). Microarray hybridization to GeneChip Rat Gene 2.0 ST arrays (Affymetrix) was performed according to the manufacturer’s protocol using the Fluidics Station 450 with the program FS450_0007. CEL files from scanned microarrays were produced with the expression console (Affymetrix).

#### Microarray Evaluation

Consistently high quality microarray data was ensured by visual inspection of scanned images for hybridization artifacts and correspondence analysis of raw and normalized microarray data. Normalizations were performed with the Quantiles method; background correction and probe set summary were achieved with Robust Microarray Average (RMA) as previously described (Buga et al., 2014). Differentially expressed genes were determined for ECS vs. sham and stroke controls vs. sham operated aged rats comparisons. The False Discovery Rate (FDR) of differential expression for the described comparisons was estimated with an empirical Bayes methodology employing lognormal normal data modeling. All analyses were performed in R version 2.14.0^1^ along with Bioconductor^2^ packages affy, EBarrays, and made4. Principle component analysis (PCA) and agglomerative hierarchical clustering (AHC) were carried out using the Rosetta Resolver Data Reduction and the 2D Cluster wizards.

#### Quantitative Real-Time PCR

For quantitative real time PCR (qPCR), we synthesized cDNA from large pools (*n* = 19–24) of total RNA with the High-Capacity cDNA reverse transcription kit (Applied Biosystems, United States). The qPCR was performed in 96-well 0.1-ml thin-wall PCR plates (Applied Biosystems) in the Step One Plus System (Applied Biosystems). Each 20 μl reaction contained 10 μl iQ SYBR Green Master Mix (Bio-Rad Laboratories, Hercules, CA, United States), 2 μl gene-specific forward and reverse primer mix (Qiagen, Alameda, CA, United States) and 8 μl pre-diluted cDNA. No template controls contained nuclease-free water instead. The cycling conditions were 3 min 95°C to activate iTaq DNA polymerase followed by 45 cycles with 30 s denaturation at 95°C, 30 s annealing at 58°C and 30 s elongation at 72°C. At the end of the amplification cycles, melting curves were used to validate PCR product specificity. All samples were amplified in triplicate. Data were analyzed using the ΔΔ*C*t method [24]. The expression levels of genes of interest were normalized to the average of expression level of the three housekeeping genes (GAPDH, HPRT1 and Ribosomal protein 19, RPL 19) from the same sample. The fold change for a gene of interest was defined as the ratio of the relative expression in the ipsilateral hemisphere (stroke lesioned, peri-infarcted or PI) to that in the sham-operated animals. All primers were provided by Eurofins, Germany.

### Statistical Analysis

The main effects of time and treatment for behavioral testing were evaluated by 2 way ANOVA followed by Dunnett’s multiple comparisons test using GraphPad software. For the analysis of histological data, we used one-way ANOVA followed by Dunnett’s multiple comparisons test for assessing the effect of treatment on the number of (i) DCX-positive cells in the hippocampus; (ii) co-localized DCX/BrdU cells in the subventricular zone and, (iii) the number of TUB/BrdU co-localized cells in the infarct core and peri-infarcted area using GraphPad software. The level of significance was set at *p* ≤ 0.05.

## Results

**FIGURE 1:**
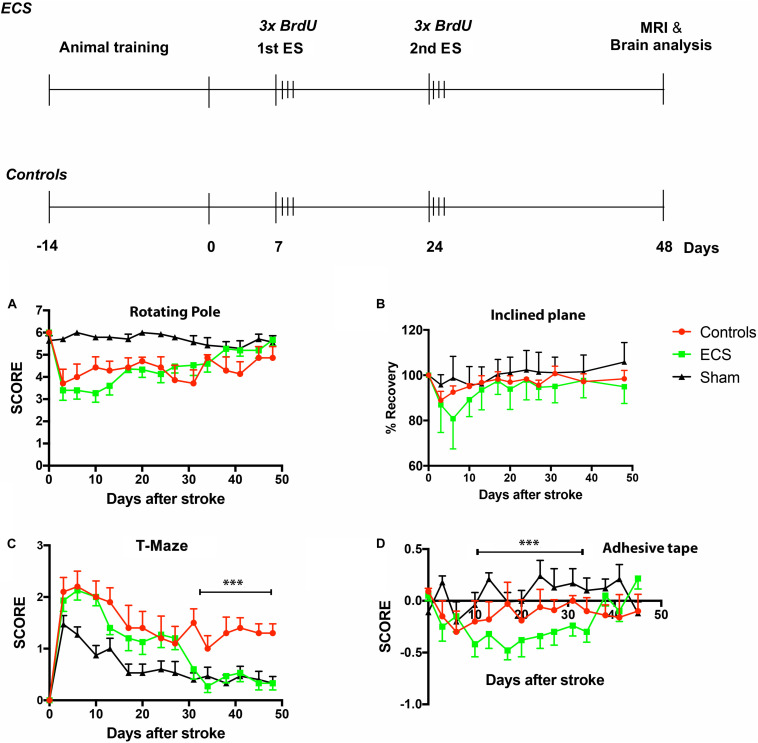
**(Upper)**: experimental design. Before surgery, rats were subjected to a variety of somatosensory, motor, learning, and memory tests (training and baseline). At 7- and 24 days after stroke, animals were stimulated by the application of ear-clip electrodes. Subsequent to survival time of 48 days, rats were subjected to MRI and the brains analyzed by immunohistochemistry. **(Lower)**: behavioral testing. There was a drop in performance shortly after the operation, part of which could be attributed to surgical stress and aging. The aged rats started recovery after a delay of 3–6 days, depending on task difficulty. Electrical stimulation had a significant beneficial effect on spatial long-term memory ***P = 0.0001 by 2-way ANOVA followed by Dunnett’s multiple comparisons test **(C)**. However, there was no beneficial effect of ES on complex sensorimotor skills like the rotating pool **(A)** or inclined plane **(B)**. Surprisingly, ES had a detrimental effect on the asymmetric sensorimotor deficit **(D)**. ***P = 0.0001 by 2-way ANOVA followed by Dunnett’s multiple comparisons test. Error bars indicate S.E.M.

The experimental design is shown in Figure 1, upper panel. For the first 3 days after the stroke, animals in all groups were fed soft pellets to facilitate nutrition. Thereafter they were permitted to eat *ad libitum.* The mortality rate was almost identical, 18% for each group, except sham-operated animals. All animals died in the first 10 days after stroke.

### Behavior

#### The Rotating Pole Task

Craniotomy in aged rats is stressful and impacts on functional recovery, especially for tests which require complex sensorimotor skills. For this reason, all animals, except the sham-operated controls, had difficulty mitigating the rotating pole in the first 3 days post-stroke. After a further decline in performance of electrically stimulated animals during the first week post-stroke, the ischemic aged rats began improvement and recovered to the pre-stroke levels by day 48 (Figure 1A). There was, however, no significant improvement in the ES group as compared to the control group.

#### Inclined Plane

Inclined plane test was used to assess complex motor and balance behavior after stroke. In aged rats, stroke severely impaired performance on the inclined plane test, and both the ES and control aged rats recovered to a limited extent during the study period (Figure 1B). Electric stimulation was not effective throughout the study period, and did not stimulate aged rats to recover any better than controls (Table 1). Recovery of sham-operated rats was complete shortly after stroke (Figure 1B).

**TABLE 1:** Analysis of differentially regulated genes.

**Sequence code**	**Gene name**	**Sequence description**	**Function**	**ES/CTRL**	***P*-value**
**Unique to ECS stimulation**					
1397833_at	LOC304554	Seizure related 6 homolog like	Seizure related	5,37	0,03
1386881_at	Igfbp3	Insulin-like growth factor binding protein 3	Neuroprotective	5,06	<0,001
1384218_at	LOC362795	Immunoglobulin G heavy chain	Chronic focal encephalitis	3,60	<0,001
1390835_at	Slc47a1	Solute carrier family 47 member 1	Brain microvessel endothelial cells	2,79	0,01
**Synergistic upregulation (upregulated both by stroke and electric stimulation)**					
1388181_at	LOC641523	Immunoglobulin delta heavy chain constant region	B cell development; unknown to stroke	11,05	<0,001
1370967_at	Cdc37	Cell division cycle protein 37	Neurogenesis, axon guidance	5,83	<0,001
1398657_at	LOC502834	Ig kappa chain V–III region MOPC 63-like	Restless legs syndrome	5,79	<0,001
1388272_at	Igh-1a	Immunoglobulin heavy constant gamma 2A	B cell development; unknown to stroke	4,48	<0,001
1374334_at	Igha	Immunoglobulin heavy constant alpha	B cell development; unknown to stroke	3,62	<0,001
1395126_at	Fcrls	Fc receptor-like S scavenger receptor	Neurodegenerative diseases	3,55	<0,001
1383163_at	Igj	Immunoglobulin J polypeptide	Ataxia teleangiectatica	3,13	<0,001
1385813_at	RGD1310251	Marginal zone B and B1 cell-specific protein	Sjogren’s syndrome	3,31	<0,001
1370394_at	IgG-2a	Gamma-2a immunoglobulin heavy chain	Development of Schizophrenia	2,37	<0,001
**Synergistic downregulation (downregulated both by stroke and electric stimulation)**					
1387680_at	Pde1b	Phosphodiesterase 1B, calmodulin-dependent	Learning and memory	−11,13	<0,001
1391938_at	Usp11	Ubiquitin specific peptidase 11	Regulation of apoptosis	−8,34	0,03
1368479_at	Drd1a	Dopamine receptor D1A	Feeding behavior; depression	−6,70	<0,001
1378899_at	Slc35d3	Solute carrier family 35 member D3	Autophagy, development	−6,11	<0,001
1370760_at	Gad1	Glutamate decarboxylase 1	Neuronal development	−2,67	0,02
1395447_at	Ermn	Ermin	Epileptic seizure; multiple sclerosis	−2,84	<0,001
1368300_at	Adora2a	Adenosine A2a receptor	Neurodegeneration	−2,34	<0,001
1369309_at	Tac1	Tachykinin precursor 1	Pain	−2,45	<0,001

#### Spatial Long-Term Memory: T-Maze

After training, aged rats learned to finish the T-maze test almost perfectly (Figure 1C). Post-stroke, T-maze performance declined sharply in all groups, including the sham-operated one. Following the second ES, however, there was a significant effect of the treatment [*F*(2,336) = 83.75, *P* < 0.0001], time [*F*(11,168) = 19.85, *P* < 0.0001], and treatment × time interaction [*F*(22,336) = 4.602, *P* < 0.0001] by 2 way ANOVA.

#### Asymmetric Sensorimotor Deficit

The adhesive tape removal test probes for differences in cutaneous sensitivity and sensorimotor integration of the forelimbs after stroke (Balseanu et al., 2014). As compared to pre-surgery values, post-stroke rats showed a significant decline in performance for the affected forelimb. By day 7, animals in the control group started recovery and reached significant recuperation by day 24 as compared to the ES group which started to recover very late, by day 34 (Figure 1D). More specifically, 2 way ANOVA analysis revealed a significant effect of treatment [*F*(2,476) = 37.14, *P* < 0.0001], time [*F*(13,238) = 3.847, *P* < 0.0001], and treatment × time interaction [*F*(26,476) = 3.371, *P* < 0.0001].

### Infarct Volume

**FIGURE 2:**
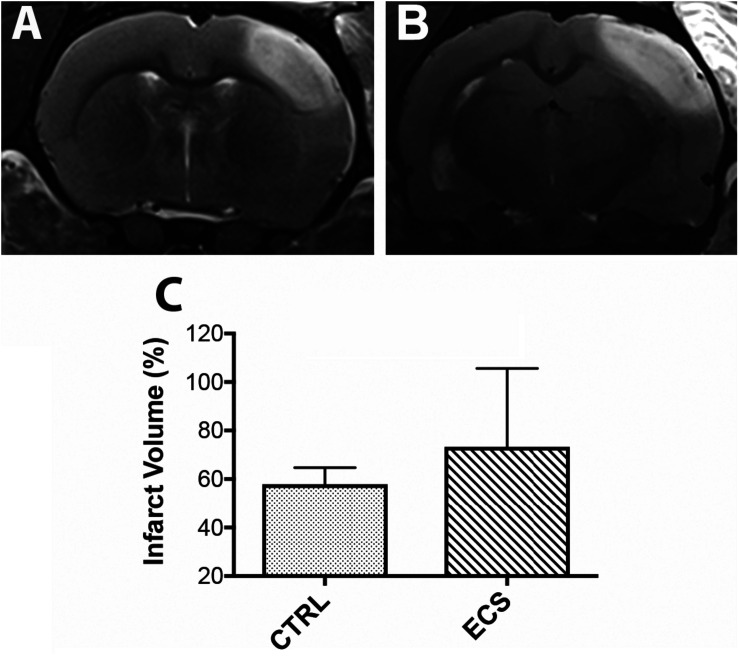
Effect of the electrical stimulation on infarct size. **(A,B)** On MRI, the ischemic lesion appeared as a hyperintense area on T2-weighted images. In some animals, the infarct volume was larger in the treatment group. However, the infarct volume was not significantly (*P* = 0.21) changed between controls and treated animals **(C)**.

Cortical infarcts as defined by the region of T2 hyperintensity for each group, are shown in Figure 2. Infarct volume was larger in the ECS group (Figure 2B) than in control rats (Figure 2A). However, the difference was not statistically significant (*P* = 0.21; Figure 2C). Noteworthy, at this post-stroke time we did not expect a significant contribution of the brain edema to the infarct volume.

### ES-Specific Long-Term Down Regulation of Gene Expression in the Perilesional Cortex

**FIGURE 3:**
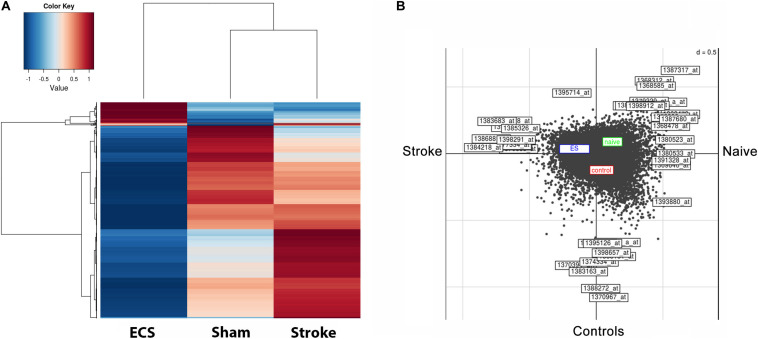
ES-specific long-term down regulation of gene expression in the perilesional cortex after stroke. The pattern of gene expression became very dissimilar by day 48 post-stroke. The heatmap shows that relative expression values clearly distinguish ES rats from their post-stroke controls **(A)**. The left-hand heatmap of ES animals subdivides expression levels into two major groups; the larger one consists of those probe sets whose expression is strongly down regulated in post-stroke animals than in controls and sham-operated ones, whereas the smaller group contains transcript clusters with higher expression than in controls and sham-operated ones. Further, we can clearly distinguish the ES effect shown in the blue box as compared with the red box for controls and green box for sham-operated animals. The effect of stroke is given as Axis 1, and the effect of the treatment as Axis 2 **(B)**.

The pattern of gene expression became very dissimilar by day 48 post-stroke. The heatmap shows that relative expression levels clearly distinguished ES rats from the corresponding controls (Figure 3A). The left-hand heatmap of ES animals is clearly divided into two major groups; a large group of transcripts those expression is strongly down regulated as compared to controls and sham-operated animals, and a much smaller group of genes displaying higher expression levels than in those in controls and sham-operated animals. The right-hand heatmap of controls also contains two major groups; one group consists of transcripts those expression is up regulated as compared both to ES- and sham-operated animals. Another group includes gene those expression was lower than in sham-operated animals but higher than in ES animals. Further, in Figure 3B, we can clearly distinguish the effect of ES, shown in the blue box, as compared with the red box for controls and green box for sham-operated animals. The effect of stroke is given as Axis 1, and the effect of the treatment is given as Axis 2.

Analysis of differentially regulated genes revealed that 4 genes were unique to ES, 9 genes were upregulated both by stroke and ES and 8 genes were downregulated both by stroke and ES. The expression of these genes was confirmed by RT-PCR (Table 1).

#### Genes Unique to Electrical Stimulation

Genes that were upregulated in the perilesional cortex of ES aged rats, included *LOC04554*, *Igfbp3*, *IgG heavy chain* and *solute carrier family 4 member 1*. Remarkably, one of these genes, *seizure related 6 homolog like* is one of the physiological substrate of the β-secretase BACE1 (β-site APP cleaving enzyme), involved in the pathophysiology of Alzheimer’s disease and a drug target (Pigoni et al., 2016). The upregulated genes included *insulin-like growth factor-binding protein 3* (*Igfbp3*) a biomarker of transient ischemic attack (Penn et al., 2018), *immunoglobulin G heavy chain* involved in chronic focal encephalitis (Baranzini et al., 2002) and *solute carrier family 47 member 1* (*Slc47a1*) expressed in brain microvessel endothelial cells (BMEC) of the blood brain barrier (Suhy et al., 2017) (Table 1).

#### Synergistic Upregulated Genes

Transcripts that were upregulated in the peri-infarcted area both by ES and lesion included genes of immunoglobulin heavy chain involved in B cells development which were knew to stroke and schizophrenia (Pandey et al., 2018) but also genes involved in *Ataxia telangiectasia* [immunoglobulin J polypeptide (IgJ)] (Castro and Flajnik, 2014; Steinel et al., 2014), Fc domain of Ig that binds to a cell surface receptor (Fcris) which is secreted by activated microglia in neurodegenerative diseases like Alzheimer‘ disease (Bisht et al., 2018) as well as Ig kappa chain V–III region MOPC 6-like (LOC50284) involved in the Restless legs syndrome (Bellei et al., 2018) (Table 1).

#### Synergistic Downregulated Genes

Downregulated genes in the perilesional cortex as compared to the corresponding cortical area of the sham-operated aged rats, included genes like *dopamine receptor D1A* (*Drd1a*) involved in feeding behavior (de Souza et al., 2020) and depression (Amiri et al., 2016), *glutamate decarboxylase* (Gad1) and *solute carrier family 5 member D* (*Slc5d3*) involved in neuronal development (Lu et al., 2014), schizophrenia (Tao et al., 2018) and autophagy (Wei et al., 2016) as well as *ubiquitin specific peptidase 11* (*Usp11*)(Table 1).

Transcripts encoding *adenosin A2a receptor* (*Adora2a*) and *ermin* (*Ermn*) have been associated with neurodegeneration (Stockwell et al., 2017), multiple sclerosis and epileptic seizures (Wang et al., 2011; Salek Esfahani et al., 2019). Finally, transcript encoding *tachykinin precursor 1* (*Tac1*) has been involved in pain-related coping responses (Huang et al., 2019) while *phosphodiesterase 1B*, *calmodulin-dependent* (*Pde1b*) was associated with the process of learning and memory (Shekarian et al., 2020).

### GO Enrichment Analysis: Genes Involved in Neuronal Development and Neuronal Growth Cone

Among the significantly downregulated genes involved in growth cone and neuronal development, we noted: *Gja1*, *Sox2*, *Map2*, *Map1b*, *p35*, *Bcl11b*, *Tmod2*, *Drd1*, *Thy1*, *Gria3*, *Nedd4*, *Kif5c*, *Negr1*, *Syn2*. Only one, coding for the BDNF receptor (*Trkb*), was upregulated. Very large decreases were noted for *Gja1* (16-fold), *Sox2* (10-fold), and *Map2* (6-fold) (Table 2).

**TABLE 2:** GO enrichment analysis: genes involved in neuronal development and neuronal growth cone.

**Sequence code**	**Sequence name**	**Sequence description**	**Fold change ECS vs. CTRL**	***P*-value**
1369640_at	Gja1	Gap junction protein, alpha 1	−16,6	<0,001
1375153_at	Sox2	SRY-box transcription factor 2	−10,5	<0,001
1368411_at	Map2	Microtubule associated protein 2	−6,6	<0,001
1369538_at	Cdk5r1	Cyclin-dependent kinase 5 (p35)	−3,7	0.006
1391948_at	Bcl11b	BAF chromatin remodeling complex	−3,6	<0,001
1369541_at	Tmod2	Tropomodulin 2	−3,4	<0,001
1368478_at	Drd1a	dopamine receptor D1A	−3,2	<0,001
1395357_at	Map1b	Microtubule associated protein 1b	−2,9	<0,001
1369651_at	Thy1	Thy1 cell surface antigen	−2,8	<0,001
1368691_at	Gria3	Glutamate receptor, ionotrophic, AMPA 3	−2,3	0.035
1375119_at	Nedd4	NEDD4 E3 ubiquitin protein ligase	−2,3	<0,001
1396154_at	Kif5c	Kinesin family member 5C	−2,1	<0,001
1387204_at	Negr1	Neuronal growth regulator 1	−2,0	<0,001
1369482_at	Syn2	Synapsin II	−2,0	<0,001
1397246_at	Ntrk2	Trkb (BDNF receptor)	2,0	<0,001

### Electrical Stimulation Led to Increased Neurogenesis in the Infarct Core

**FIGURE 4:**
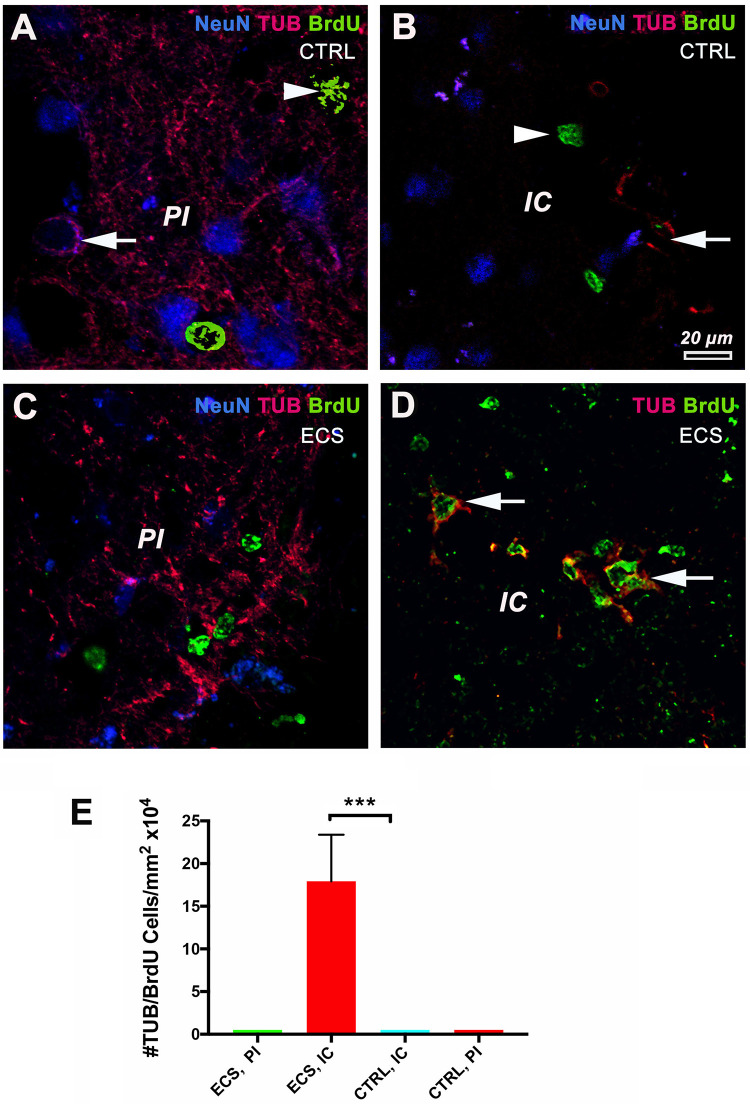
Electrical stimulation led to increased neurogenesis in the infarct core. In control animals there were many co-localized NeuN/Tubulin beta III cells in the perilesional area (**A**, arrow). However, we could not detect co-localized BrdU/Tubulin beta III cells in the infarct core itself (**B**, arrowhead). Similarly, there was no clear colocalization of BrdU nuclei with Tubulin beta III in the perilesional area of ES animals **(C)**. Noteworthy, the number of BrdU-labeled Tubulin beta III cells in the infarct core of ES animals showed a significant 18-fold increase over controls (**D**, arrows). **(E)** Number of co-localized TUB/BrdU cells in the infarct core. ***P = 0.0001 by one-way ANOVA followed by Dunnett’s multiple comparisons test. PI, peri-infarct; IC, infarct core.

To assess whether ES led to an increased number of newborn cells in the ischemic area, we performed a triple immunostaining using neuronal markers (NeuN, Tubulin beta III) and a marker of cell proliferation, Brdu (Liu et al., 2020) (Figure 4). In control animals there were many co-localized NeuN/Tubulin beta III cells in the perilesional area (Figure 4A, arrow). However, we could not detect co-localized BrdU/Tubulin beta III cells in the infarct core itself (Figure 4B, arrowhead). Similarly, there was no clear colocalization of BrdU nuclei with Tubulin beta III in the perilesional area of ES animals (Figure 4C). However, the number of BrdU-labeled Tubulin beta III cells in the infarct core of ES animals showed a significant 18-fold increase {*P* = 0.0001; [*F*(DFn, DFd) = 9.611 (3,76)]} by one-way ANOVA (Figure 4E) over the perilesional area of controls (Figure 4D, arrows).

### EC Stimulation Led to Increased Neurogenesis in the Hippocampus

**FIGURE 5:**
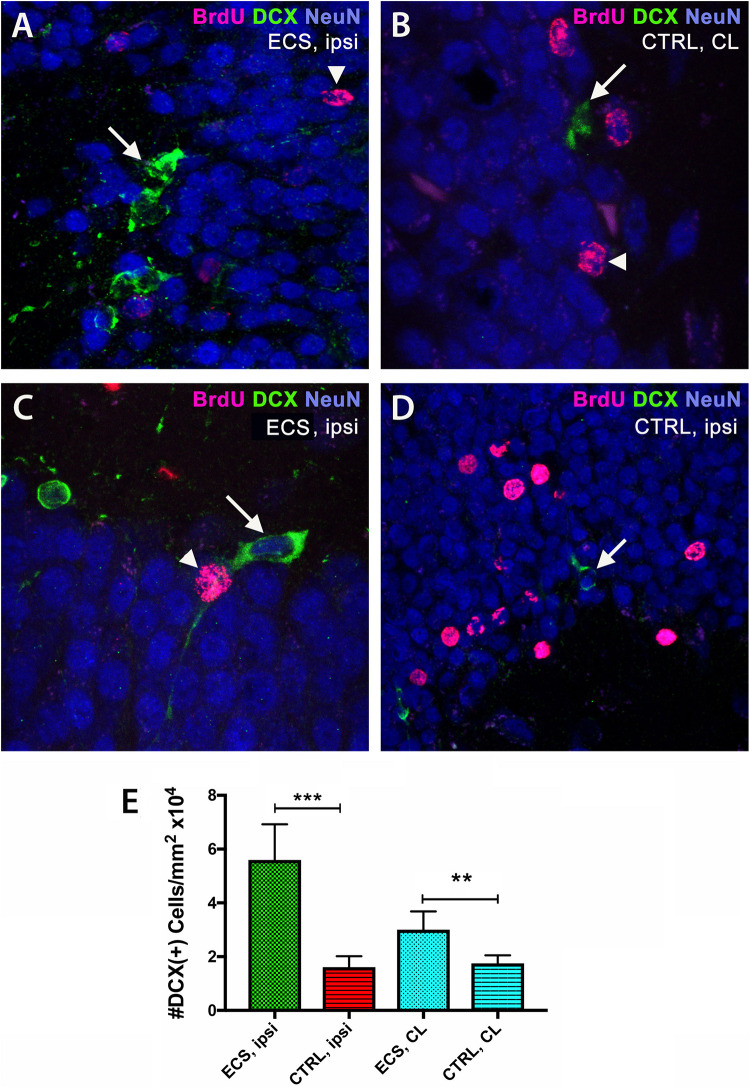
Electrical stimulation led to increased neurogenesis in the hippocampus. By double immunofluorescence, in the ipsilateral hippocampi of ES rats we noted vigorous (threefold) increase in the number of doublecortin cells **(A)** over the ipsilateral side of controls **(C)**. Stroke injury did increase the number pf BrdU^+^ cells both in the ipsilateral hippocampus **(D)** and the contralateral hippocampus **(B)** of controls. Of note, in the contralateral side, the effect of stimulation on the number of BrdU-positive nuclei was less evident (1.8-fold) **(E)**. ***P = 0.0001 by one-way ANOVA followed by Dunnett’s multiple comparisons test. ES, electrostimulation; ipsi, ipsilateral; CL, contralateral.

Next, we counted the numbers of the early neuronal marker doublecortin (DCX) by immunofluorescence in the hippocampus of aged rats after focal ischemia. In the ipsilateral hippocampi of ES rats we noted vigorous increase [threefold; *P* = 0.0001; *F*(3,76) = 110.5] by one-way ANOVA (Figures 5A,E), over the ipsilateral side of controls (Figure 5C). Of note, stroke injury did increase the number pf BrdU^+^ cells both in the contralateral (Figure 5B) and the ipsilateral hippocampus of controls (Figure 5D). However, in the contralateral side, the effect of stimulation on the number of DCX-positive cells, although significant, was less evident (1.8-fold; *P* = 0.001) by Dunnett’s multiple comparison test (Figure 5E).

### EC Stimulation Led to Increased Neurogenesis in the Subventricular Zone

**FIGURE 6:**
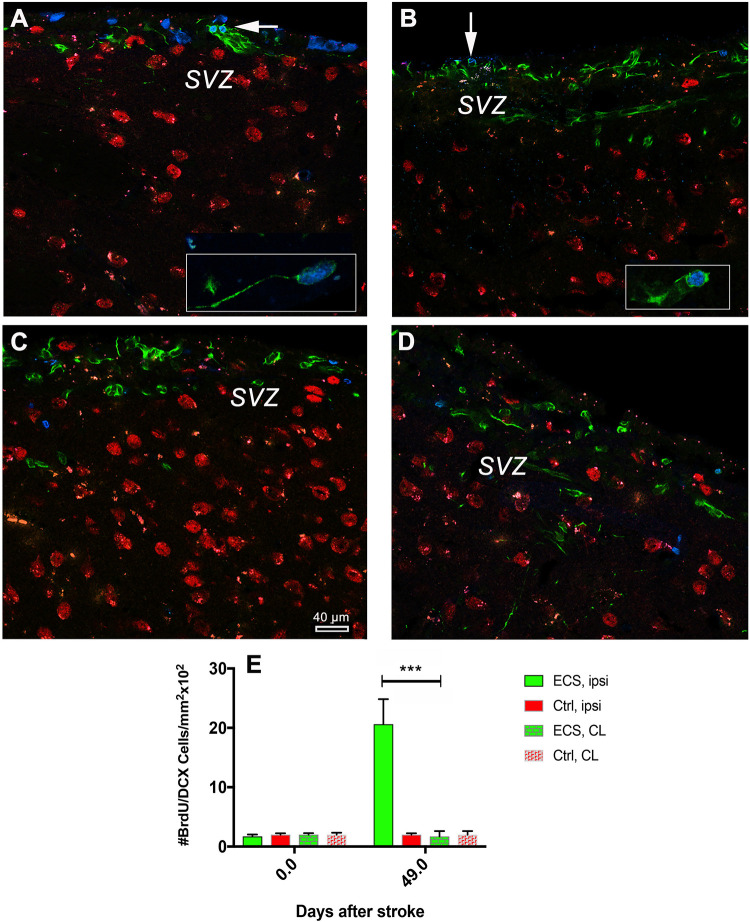
Electrical stimulation led to increased neurogenesis in the subventricular zone. Analysis of the numbers of co-localized BrdU/DCX cells in the subventricular zone revealed a significant increase (18-fold in the number of BrdU/DCX+ cells in the ipsilateral hemisphere of ES animals) (**A**, and inset) as compared with the contralateral hemisphere of the same animals (**B**, and inset; **E**). In control animals, despite of large numbers of DCX and BrdU-positive cells, we could not detect clear co-localized BrdU/DCX cells both in the ipsilateral and contralateral hemisphere (**C** vs. **D**). ***P = 0.0001 by one-way ANOVA followed by Dunnett’s multiple comparisons test.

Cerebral ischemia stimulates cell proliferation in the SVZ and multiply the number of migrating neurons which express DCX that into the nearby lesioned area (Arvidsson et al., 2002; Parent et al., 2002). Given the time elapsed after the first and second stimulation and the time required for neuronal differentiation of DCX cells, we aimed to find out how many cells are still labeled with a BrdU nucleus at 48 days post-stroke. The analysis of the numbers of co-localized BrdU/DCX in the ipsilateral subventricular zone revealed a significant increase {ninefold; *P* = 0.0001; [*F*(3, 76) = 110.5]} by one-way ANOVA, in the number of BrdU/DCX+ cells in the ipsilateral hemisphere of ES animals (Figure 6A) as compared with the contralateral hemisphere of the same animal (Figures 6A vs. 6B) and Figure 6E. In control animals, despite of large numbers of DCX and BrdU-positive cells, we could not detect clear co-localized BrdU/DCX cells both in the ipsilateral and contralateral hemisphere (Figures 6C vs. 6D).

## Discussion

Despite remarkable advances in cell therapy for stroke, stem cell-based tissue replacement has not been achieved yet, stimulating the search for alternative strategies for brain self-repair using the neurogenic zones of the brain, the dentate gyrus and the subventricular zone (Liu et al., 2013). However, it is not clear whether increased neurogenesis improves neurobehavioral recovery or aids brain repair. In this study we report that electrical stimulation (ES) of post-stroke aged rats led to a considerable increase in the number of Tubulin beta III in the infarcted area and a robust increase in newly born DCX cells in the dentate gyrus and SVZ of the infarcted hemisphere. Behaviorally, ES had a beneficial effect on spatial long-term memory (T-maze) but not on the rotating pool or inclined plane, both tests requiring complex sensorimotor skills. However, ES had a detrimental effect on the asymmetric sensorimotor deficit.

Neural stem cells (NSCs) persist in the adult mammalian brain in two specific regions, the dentate gyrus of the hippocampus, and the subventricular zone (Quiñones-Hinojosa et al., 2006) throughout life. The subventricular zone (SVZ) is involved in brain regeneration by continuously generating new neuronal precursors from embryonic radial glia cells (Fuentealba et al., 2015). The human SVZ also acts as a neurogenic niche generating new neurons (Quiñones-Hinojosa et al., 2006). Much like in rodents, in humans, SVZ-derived neuroblasts migrate via rostral migratory stream (RMS) into the olfactory bulb of infants (Guerrero-Cázares et al., 2011).

In the adult brain, traumatic brain injury and ischemia greatly stimulate neurogenesis in the SVZ, generating calretinin-positive neurons in regions nearby SVZ/RMS such as the striatum (Jankovski et al., 1998; Liu et al., 2009). Similarly, the electroconvulsive therapy (ECT), a highly effective treatment of severe depression, may induce plastic changes in frontal limbic circuitry by stimulating neurogenesis in the hippocampus, dentate gyrus and subventricular zone (Buga et al., 2012; Inta et al., 2013). However, all ECT stimulus parameters should be considered explicitly in dosage since summary metrics like charge do not uniquely determine the stimulus and its physiological effects. In addition, ECT electrode configuration should be carefully considered. For example, brief-pulse bifrontotemporal ECT, although highly effective with a 70–80% response rate, it may result in significant cognitive side effects (Sackeim et al., 2000, 2008; Lisanby, 2007; Peterchev et al., 2010).

The mechanisms underlying the beneficial effects of non-invasive brain electric stimulation are not fully understood. Numerous studies have revealed that brain stimulation of the ventromedial prefrontal cortex in middle aged rats, enhanced short-term memory in the novel-object recognition task (Liu et al., 2015), improved depressive behavior (Zhang et al., 2001) and restored the number of neuronal precursors after spinal cord injury (SCI) (Becker et al., 2010). Likewise, transcranial direct current stimulation also stimulates neurogenesis in mice (Pikhovych et al., 2016) and in a rat model of stroke along with oligodendrocyte precursors recruitment (Braun et al., 2016).

The abnormal brain electrical activity may result in a robust increase in the number of neuronal precursors in the adult dentate gyrus (Parent et al., 1997). In addition, in previous work we have shown that the time required for neuronal maturation after stimulating neurogenesis in the subventricular zone and hippocampus the brain is 24 days (Buga et al., 2012). For this reason, we applied the stimulus at 7- and 24 days after stroke. Using this time frame, we also avoided the fulminant inflammatory reaction to brain injury in aged brains (Popa-Wagner et al., 2007).

### ES-Specific Long-Term Down Regulation of Cortical Gene Expression After Stroke

It is not clear how to corelate the changes in gene expression that would support the increased in neurogenesis and the beneficial effects on spatial long-term memory (T-maze). On one hand, there was an upregulation of several genes expressed in activated microglia in neurodegenerative diseases like Alzheimer’ disease (Bisht et al., 2018). Indeed, non-invasive brain electrical stimulation leads to microglia activation in brain slices tempting to speculate that M2 microglia might support plasticity relevant for neurorehabilitation and network re-organization in the diseased brain (Gellner et al., 2016). On the other hand, there were genes that were unique to ES including *seizure related 6 homolog* like, one of the physiological substrate of the β-secretase BACE1 (Pigoni et al., 2016) and *Igfbp3* which has been shown to have a neuroprotective effect by modulating the bioavailability of insulin-like growth factor-I (IGF-I) (Mangiola et al., 2015). Furthermore, serum IGFBP3 could represent a biomarker for post-stroke outcome and functional recovery (Ebinger et al., 2015).

In the clinic, ECT is widely used for the treatment of major depression, as well as for treatment of catatonic schizophrenia and acute mania (Sackeim et al., 1983). Indeed, among the downregulated genes, we identified the gene coding for *dopamine receptor* D1A (*Drd1a*) involved in depression (Amiri et al., 2016). Also downregulated were most of the genes involved in growth cone and neuronal development suggesting an additional mechanisms of the ETC treatment in schizophrenia patients.

Behaviorally, the aged rats could also have benefitted from the downregulation of genes involved in schizophrenia like solute carrier family 5member D (*Slc5d3*) (Tao et al., 2018), or transcripts encoding *adenosin A2a receptor* (Adora2a) and ermin (*Ermn*) have been associated with neurodegeneration (Stockwell et al., 2017), multiple sclerosis and epileptic seizures (Wang et al., 2011; Salek Esfahani et al., 2019).

## Conclusion

Electrical stimulation of post-stroke aged rats led to an improved functional recovery of spatial long-term memory but not on the rotating pool or inclined plane, both tests requiring complex sensorimotor skills. Surprisingly, ES had a detrimental effect on the asymmetric sensorimotor deficit. Histologically, there was a robust increase in the number of newly born DCX cells in the dentate gyrus and SVZ of the infarcted hemisphere and a considerable presence of neuronal marker, tubulin beta III in the infarcted area. ES was associated with a long-term down regulation of cortical gene expression after stroke and enhanced recuperation after cerebral ischemia in aged rats suggesting that gene expression in the peri-infarcted area may not be linked to electrical stimulation induced-neurogenesis in the subventricular zone and hippocampus.

## Data Availability Statement

The original contributions presented in the study are included in the article/supplementary material, further inquiries can be directed to the corresponding author/s.

## Ethics Statement

The animal study was reviewed and approved by Animal Experimentation Ethics Board of the State of Mecklenburg-Vorpommern.

## Author Contributions

AP-W, DH, and MS: conceptualization. AB, MG, and L-RP: methodology. AP-W, DH, and DG: formal analysis. AP-W and DG: writing – original draft. AP-W and DH: supervision. AP-W: project administration and funding acquisition. All authors contributed to the article and approved the submitted version.

## Conflict of Interest

The authors declare that the research was conducted in the absence of any commercial or financial relationships that could be construed as a potential conflict of interest.

## Footnotes

1 www.r-project.org

2 www.bioconductor.org
